# The Effect of Surgical Aortic Valve Replacement on Arterial Stiffness: Does the Valve Type Matter?

**DOI:** 10.3390/jpm14050509

**Published:** 2024-05-11

**Authors:** Evangelia Sigala, Dimitrios Terentes-Printzios, Vasiliki Gardikioti, Nikolaos G. Baikoussis, Nikolaos Koumallos, Andreas Katsaros, Vasileios Lozos, Ilias Kouerinis, Konstantinos Triantafillou, Konstantinos Filis, Konstantinos Tsioufis, Charalambos Vlachopoulos

**Affiliations:** 1 First Department of Cardiology, Hippokration Hospital, 11527 Athens, Greece; 2 Department of Cardiology, Tzaneio General Hospital, 18536 Piraeus, Greece; 3 Department of Cardiac Surgery, Hippokration Hospital, 11527 Athens, Greecekoumallosn@yahoo.gr (N.K.);; 4 First Department of Surgery, Hippokration Hospital, 11527 Athens, Greece; kfilis@hotmail.com

**Keywords:** aortic stenosis, aortic stiffness, arterial biomarkers, pulse wave velocity, SAVR

## Abstract

**Background:** Despite the increasing use of transcatheter aortic valve procedures, many patients still require surgical aortic valve replacement (SAVR). Assessing arterial properties in patients undergoing SAVR for aortic valve stenosis can be challenging, and the existing evidence is inconclusive. Our study aimed to investigate the impact of SAVR on vascular stiffness and the quality of life, as well as the different effects of valve type on arterial properties. **Methods:** We included 60 patients (mean age 70.25 ± 8.76 years, 65% men) with severe symptomatic aortic stenosis who underwent SAVR. Arterial stiffness (cfPWV, baPWV) and vascular parameters (AIx@75, central pressures, SEVR) were measured at baseline, pre-discharge, and 1-year post-operation. The QOL was assessed using the generic questionnaire—short-form health survey 36 (SF-36) pre-operatively and at 1 year. **Results:** Post-SAVR, cfPWV increased immediately (7.67 ± 1.70 m/s vs. 8.27 ± 1.92 m/s, *p* = 0.009) and persisted at 1 year (8.27 ± 1.92 m/s vs. 9.29 ± 2.59 m/s, *p* ≤ 0.001). Similarly, baPWV (n = 55) increased acutely (1633 ± 429 cm/s vs. 2014 ± 606 cm/s, *p* < 0.001) and remained elevated at 1 year (1633 ± 429 cm/s vs. 1867 ± 408 cm/s, *p* < 0.001). Acute decrease in Alx@75 (31.16 ± 10% vs. 22.48 ± 13%, *p* < 0.001) reversed at 1 year (31.16 ± 10% vs. 30.98 ± 9%, *p* = 0.71). SEVR improved (136.1 ± 30.4% vs. 149.2 ± 32.7%, *p* = 0.01) and persisted at 1 year (136.1 ± 30.4% vs. 147.5 ± 30.4%, *p* = 0.01). SV had a greater cfPWV increase at 1 year (*p* = 0.049). The QOL improved irrespective of arterial stiffness changes. **Conclusions:** After SAVR, arterial stiffness demonstrates a persistent increase at 1-year, with valve type having a slight influence on the outcomes. These findings remain consistent despite the perceived QOL.

## 1. Introduction

Aortic valve stenosis (AVS) is the most common valvular disease in the elderly [1], with an increasing prevalence and associated morbidity and mortality [2,3]. Progression of the disease to a degenerative form is due to a combination of factors including lipoprotein deposition, chronic inflammation, and active leaflet calcification [4]. In addition, AVS involves vascular changes and adaptive processes in the left ventricle (LV). These are additional components that contribute to the pathogenesis of degenerative AVS and play a role in the development of complications such as hypertension, coronary artery disease, diabetes, and stroke [1,4]. The complexity of AVS and its impact on both the LV and the vessels make managing the disease challenging. While transcatheter aortic valve implantation (TAVI) is gaining popularity and is increasingly being used in clinical practice [5,6], SAVR remains a preferred option for many patients with AVS, particularly those who are younger and at lower surgical risk [7,8,9].

Arterial stiffness is a hallmark of vascular aging that shares common pathophysiological mechanisms with AVS and has been widely associated with adverse cardiovascular events [10]. Increased arterial stiffness is manifested in various pathologic states, including hypertension, atherosclerosis, cerebrovascular disease, and other chronic diseases [10]. Carotid-to-femoral pulse wave velocity (cfPWV) and brachial–ankle pulse wave velocity (baPWV) are considered to be the gold standard for assessing arterial stiffness according to the ESH Guidelines 2023 [11]. The physiological range of values for arterial stiffness is essential for understanding its clinical significance. Elevated cfPWV or baPWV values have been associated with a worse prognosis for both short-term and long-term survival in various populations, particularly in hypertensive individuals [11,12]. For people aged 50 to 60 years, a threshold for cfPWV abnormality is set at values above 10 m/s [11]. In addition, studies have shown that a reduction in cfPWV may be associated with improved outcomes in patients with hypertension or on dialysis, highlighting the clinical relevance of monitoring this parameter [11,13]. Notably, in patients with moderate to severe AVS, a cfPWV < 10 m/s was associated with lower event-free survival than in patients with a cfPWV ≥ 10 m/s [14].

When AVS is present, increased arterial stiffness may further burden the cardiovascular system, potentially leading to LV deterioration and poor prognosis [9,15]. After SAVR, the reduction in afterload forces is expected to result in reverse LV remodelling and reduced ventricular hypertrophy [16]. However, the magnitude of functional benefits from LV surgery could be mitigated by an excessive increase in aortic stiffness [16,17]. Also, the LV and AV remodeling processes may subsequently affect hemodynamics in the CV system [18,19]. These changes may influence the transmission of pressure waves along the arterial tree and thereby affect arterial stiffness measured with cfPWV [20]. Nevertheless, studies examining the effects of AVR on arterial stiffness are heterogeneous and limited in the literature, making it difficult to unequivocally answer important questions such as the exact timing of fluctuations or decision and treatment options [17]. Although vascular biomarkers provide complementary information, their prolonged trajectory after SAVR is yet to be studied.

Considering the significant roles of arterial stiffness [14,15,16,17,18,19,20,21] and central hemodynamics [22,23,24] in cardiovascular disease prognosis, this study aims to evaluate the effect of SAVR on arterial elastic properties using multiple vascular parameters (cfPWV, baPWV, and central hemodynamics) over a 12-month follow-up. Furthermore, this study aims to investigate the potential effects of different valve types (mechanical-MV, sutureless-SV, bioprosthetic-BV) on arterial properties and assess how changes in arterial stiffness after SAVR impact patients’ QOL.

## 2. Materials and Methods

### 2.1. Study Population

We enrolled 60 patients (mean age 70.8 ± 8.4 years) with severe symptomatic AVS since January 2016 and their follow-up was completed in May 2019. Severe AVS was defined with echocardiographic examination based on recommendations from the current international guidelines: an aortic valve area (AVA) < 1.0 cm^2^ (or an aortic valve area index < 0.6 cm^2^/m^2^, mean gradient (MG) > 40 mmHg or peak aortic valve velocity > 4.0 m/s) [9]. Supplementary Materials S1 outlines the criteria of exclusion. This research was conducted in accordance with the Declaration of Helsinki [25], and Institutional Research Ethics Committee of our research center approved the protocol (approval code: 6382-19/04/2018). Written informed consent was obtained from all participants.

Most patients were referred to our center for surgical intervention due to the progression of AV severity disease and the onset of symptoms such as exertional angina, syncope, and dyspnea. During the initial visit, a recording of sociodemographic information, medical history, cardiovascular disease risk factors, and QOL status was conducted through interviews with all patients. QOL was assessed using the validated generic questionnaire—short-form health survey 36 (SF-36). The process of QOL evaluation is explained in Supplementary Materials S2 [26,27,28,29]. Additionally, physical examination, laboratory tests, ultrasound examination, and baseline arterial stiffness values were recorded.

### 2.2. Study Design

**Figure 1:**
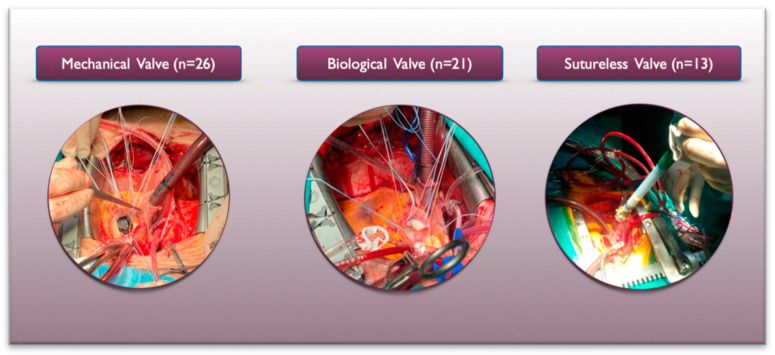
Type of valve prosthesis.

The analysis included patients who underwent isolated SAVR or SAVR with cardiopulmonary bypass grafting (CABG) and returned for follow-up after one year. With AVS as the primary cause, patients received an MV, BV, or SV prosthesis depending on international criteria and their individual condition (Figure 1) [7]. The study design consisted of 3 different time points: baseline, at discharge (4–9 days after SAVR), and at 1 year. After the baseline assessment was performed in detail, the same examination was repeated at discharge (4–9 days after SAVR, no QOL assessment) and at 1 year.

The measurements were taken under standardized conditions [30], and the laboratory where the measurements were conducted was reliable with good measurement repeatability. The same researchers performed the measurements throughout the study. The treatment of patients receiving periprocedural beta-blockers or antihypertensive therapy was routinely discontinued before or after SAVR, and surgeon instructions were followed on an individual basis (Supplementary Materials S3).

### 2.3. Assessment of the Arterial Elastic Properties, Peripheral and Central Blood Pressures, and Wave Reflection Indices

Arterial elastic properties were evaluated using 2 non-invasive methods, cfPWV and baPWV. Both indices are established parameters of aortic stiffness with demonstrated predictive value [21,24,31,32,33,34,35]. For the measurement of cfPWV and baPWV, the validated Complior (Artech Medical, Paris, France) and BP-203RPE III (VP-1000, Omron Colin, Japan) devices were used accordingly [21]. The difference between two measured points (suprasternal notch to the femoral artery and carotid artery to the suprasternal notch) was the final definition of distance for calculating cfPWV. To measure baPWV, pressure cuffs were wrapped around the brachium and ankles. Patients with an ankle–brachial index (ABI) less than 0.9 (n = 5) were excluded from the analysis to avoid inaccuracies in baPWV measurements.

To measure peripheral blood pressures, the Omron Colin Japan automated device BP-203RPE III (VP-1000) was used on the right upper arm over the brachial artery. Central pressures and wave reflection parameters (aortic pressures, augmentation index corrected for heart rate- alx@75, subendocardial viability ratio-SEVR) are widely recognized as independent predictors of cardiovascular disease and mortality, and they signify a composite assessment of arterial stiffness [23]. SEVR reflects the degree of endocardial ischemia and provides information about myocardial perfusion [32,36]. Measurements of these indices were taken with validated equipment: SphygmoCor, AtCor Medical from Sydney, Australia (Supplementary Materials S4).

### 2.4. Laboratory Tests

Upon patient admission before surgery, venous blood was collected to assess hemoglobin, glucose, serum creatinine, and lipidemic profiles. The Modification of Diet in Renal Disease (MDRD) equation was used to calculate the estimated glomerular filtration rate (eGFR).

### 2.5. Statistical Analysis

A power analysis was performed before the study to determine the desired sample size. A power analysis of variance for repeated measurements (repeated ANOVA) yielded a desired sample size of n = 60 with a statistical power of 80% to detect an increase of 0.5 m/s in cfPWV immediately and 1 year after SAVR [32,37]. Based on the results of the analysis, 60 patients were included and completed the follow-up of our study. Continuous variables are expressed as means ± S.D. Normality was tested by using the Kolmogorov–Smirnov criterion. Normally distributed variables are presented as mean ± constant standard deviation. Non-normally distributed variables are presented as median (25–75th percentile).

A univariate ANOVA was used to assess population changes from the baseline to post-SAVR and the 1-year follow-up. Between-group comparisons for categorical or non-continuous variables were performed using Kruskal–Wallis rank tests or the chi-square test. Pearson’s correlation coefficient was used to analyze correlations between the study variables. Multivariable analysis was performed by using either analysis of covariance (ANCOVA) or linear multiple regression analysis and logistic regression analysis, as appropriate. Bonferroni correction was used to compare each of these timepoints when an ANOVA or ANCOVA revealed a significant difference between them. The mean SF-36 difference of the eight domains was calculated and compared to the change in carotid-femoral pulse wave velocity (ΔcfPWV) from pre-SAVR to 1-year post-SAVR.

Statistical analysis was performed with SPSS program, version 25 (SPSS Inc., Chicago, IL, USA). *p* values less than 0.05 were considered to be indicative of statistical significance.

## 3. Results

### 3.1. Vascular Biomarkers and Subject Periprocedural Characteristics

The characteristics of the study population are presented in Table 1. A majority of them, 65%, were male, and their mean age was 70.8 ± 8.5 years. Most patients had a low surgical risk (Euroscore II 2.46 ± 2.28%) and the ejection fraction was preserved (55 ± 9%). Regarding their vascular characteristics, baseline cfPWV and baPWV were 7.67 ± 1.70 m/s and 1633.36 ± 429 cm/s, respectively (Table 2). The Alx@75 index was 31.16 ± 10.22% and SEVR was 136.16 ± 30.42% before SAVR (Table 2). Antihypertensive treatment remained largely unchanged during the procedure (Supplementary Materials S3). Post-operative atrial fibrillation (POAF) (n = 11, 19%) was the most frequent complication after SAVR (Supplementary Materials S5) and all patients received immediate pharmacological treatment and returned to sinus rhythm before discharge. Τhe median length of hospital stay was 6 days (interquartile range: 5–7 days).

### 3.2. Effects of SAVR on Arterial Stiffness across the Entire Patient Cohort

#### 3.2.1. cfPWV

**Figure 2:**
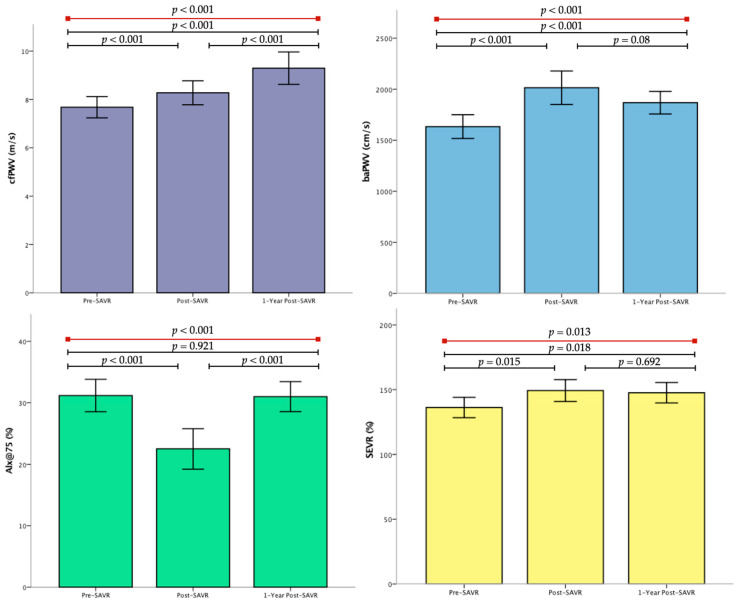
Change in vascular biomarkers post-SAVR. *p* values were obtained by analysis of variance and paired-samples analysis. cfPWV, carotid-femoral pulse wave velocity; baPWV, brachial–ankle pulse wave velocity; SEVR, subendocardial viability ratio; AIx@7F5, augmentation index corrected for heart rate.

Over the course of the follow-up, there was a significant increase in cfPWV (*p* < 0.001, Figure 2). Comparing baseline values with values at discharge, it was found that there was a statistically significant increase in cfPWV (7.67 ± 1.70 m/s vs. 8.27 ± 1.92 m/s, *p* = 0.009). Even after one year of SAVR, cfPWV was persistently elevated compared to pre- and post- intervention assessments (7.67 ± 1.70 m/s vs. 9.29 ± 2.59 m/s, *p* < 0.001 and 8.27 ± 1.92 m/s vs. 9.29 ± 2.59 m/s, *p* < 0.001). As shown in the multivariable regression analysis (Supplementary Materials S6), the change in cfPWV (ΔcfPWV) was associated with baseline cfPWV and change in echocardiographic peak aortic valve velocity from baseline to 1-year after-SAVR.

ΔcfPWV was not different when compared in groups with and without baseline risk factors and comorbidities, including hypertension (*p* = 0.42), dyslipidemia (*p* = 0.85), diabetes mellitus (*p* = 0.49), and CAD or CABG (*p* = 0.48). Patients diagnosed with CAD and who underwent CABG had the following values: at baseline, cfPWV for those who did not have CAD and did not undergo CABG was 7.82 ± 1.67 vs. 7.35 ± 1.77 m/s for those who were diagnosed with CAD and underwent CABG. Accordingly, their post measurements were 8.16 ± 1.76 vs. 8.51 vs. 2.26 m/s and their measurements one year after SAVR were 9.30 ± 2.55 vs. 9.27 ± 2.74 m/s. Their difference in ΔcfPWV was 1.47 ± 2.45 vs. 1.91 ± 1.87 m/s.

#### 3.2.2. baPWV

Equally, in the same period of 1-year follow-up, baPWV (Figure 2) showed a significant increase (*p* < 0.001). Immediately after SAVR, baPWV increased significantly (1633 ± 429 cm/s vs. 2014 ± 606 cm/s, *p* < 0.001) and remained increased 1 year after SAVR compared to measurements before surgery (1633 ± 429 cm/s vs. 1867 ± 408 cm/s, *p* < 0.001). However, there was a non-significant trend for higher values compared to post-SAVR measurements (*p* = 0.08). Further analysis revealed that baPWV showed a correlation with cfPWV in the measurements before SAVR (*p* = 0.01, r = 0.339), and this correlation became even more significant in the measurements one year after SAVR (*p* < 0.001, r = 0.571). Finally, there was a positive correlation between ΔbaPWV and ΔcfPWV (*p* = 0.005, r = 0.355).

#### 3.2.3. Wave Reflections

Alx@75, which showed significant fluctuations as well (*p* < 0.001, Figure 2), exhibited a decrease compared to the baseline (31.16 ± 10.22% vs. 22.48 ± 12.71%, *p* < 0.001) and did not change at 1 year (31.16 ± 10.22% vs. 30.98 ± 9.47%, *p* = 0.921). On the other hand, SEVR increased shortly after SAVR (136.16 ± 30.42 vs. 149.25 ± 32.74, *p* = 0.015) and kept increasing during the follow-up (with significant difference between measurements, *p* = 0.013) in comparison to the baseline values (136.16 ± 30.42 vs. 147.50 ± 30.48, *p* = 0.018). The results of the central pressure measurements are shown in Supplementary Materials S7 and S8.

### 3.3. QOL in Correlation with Arterial Stiffness

The QOL showed significant improvement after SAVR, as shown by the results of the SF-36 instrument (Supplementary Materials S9). The physical summary score (PCS) increased from 56.50% preoperatively to 82.50% at 1 year (*p* < 0.001). Similarly, the mental health component summary (MCS) showed improvement: 55.50% vs. 80%, *p* < 0.001). The mean differences in SF-36 domain scores between pre-SAVR and 1 year after SAVR, as well as the changes in carotid–femoral pulse wave velocity (ΔcfPWV), are also presented in Supplementary Materials S9. However, no association was found between the mean differences in the SF-36 domains and changes in ΔcfPWV. The difference (Δ) in PCS between pre-SAVR and 1-year follow-up was +24, while in MCS it was +25. Interestingly, no correlation was observed between ΔcfPWV and the differences in QOL from before surgery to 1 year later (Supplementary Materials S9).

### 3.4. Effects of SAVR on Arterial Stiffness According to Prosthesis Valve Type 

**Figure 3:**
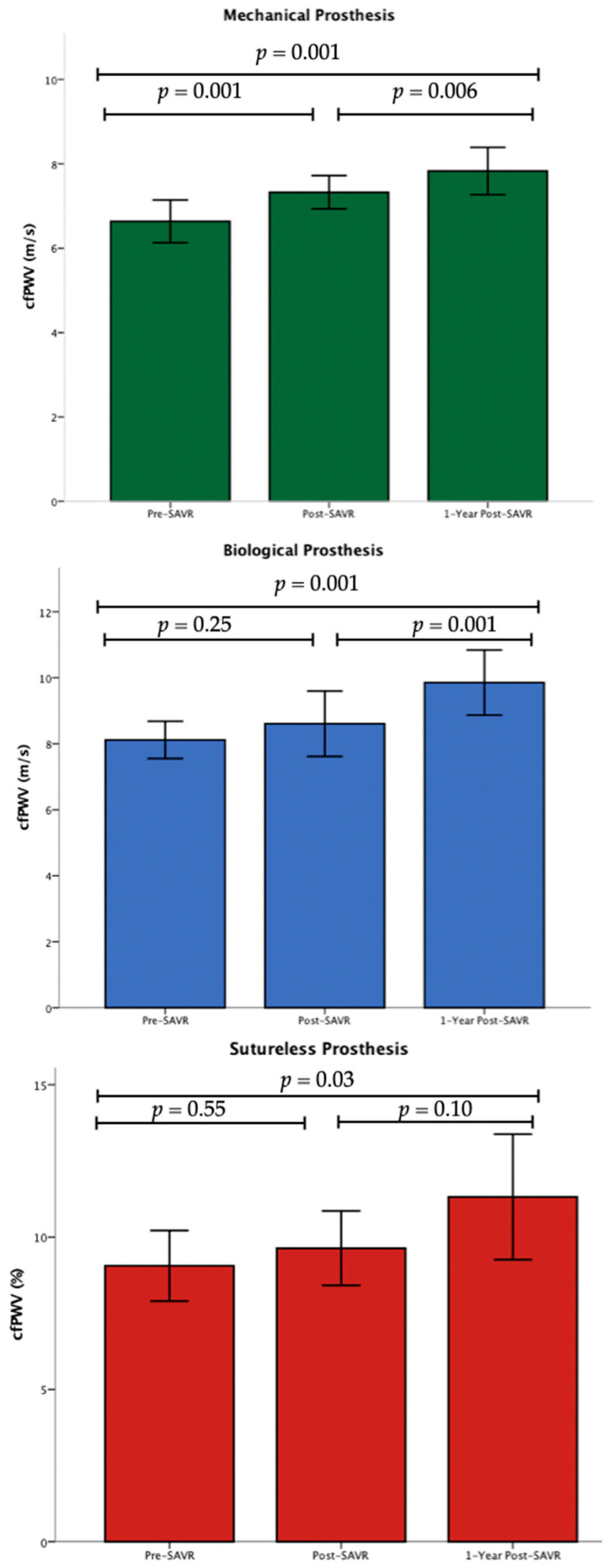
Changes in cfPWV to valve type at 3 time points (N = 60).

The impact of different valve types on arterial stiffness following SAVR is analyzed in Table 3. The baseline and periprocedural characteristics of SAVR patients according to prosthesis valve type and correlations of each valve type with ΔcfPWV are shown in Table 4. Immediately after SAVR, there was an increase in cfPWV in each valve-type group. However, due to the small number of subjects in the non-MV groups and despite the numerical increase in cfPWV, only the changes in the MV group were significant (MV 6.63 ± 1.25 m/s vs. 7.32 ± 0.97 m/s, *p* = 0.001; BV 8.11 ± 1.24 m/s vs. 8.60 ± 2.18 m/s, *p* = 0.25; SV 9.05 ± 1.91 m/s vs. 9.63±2.01 m/s, *p* = 0.55, Figure 3. After 1 year, cfPWV was significantly increased in all groups (MV 6.63 ± 1.25 m/s vs. 7.82 ± 1.38 m/s, *p* < 0.001; BV 8.11 ± 1.24 m/s vs. 9.85 ± 2.16 m/s, *p* < 0.001; SV 9.05 ± 1.91 m/s vs. 11.31 ± 3.4 m/s, *p* = 0.03). The SV group showed the greater increase (+2.25 ± 3.70 m/s) compared to other groups (BV group +1.73 ± 1.68 m/s, MV group +1.19 ± 1.72 m/s, Figure S5 in Supplementary Materials S10). Multivariate analysis showed that patients with SV had higher long-term cfPWV values compared to other types of valves, regardless of age, gender, and peripheral systolic blood pressure at 1 year (*p* = 0.049, Supplementary Materials S6). The changes in baPWV one year after SAVR for each valve type are summarized in Table 3. Specifically, the MV group showed a change of +208.16 ± 284.30 cm/s in baPWV, the BV group showed a change of +207.88 ± 404.85 cm/s, and the SV group showed a change of +319.38 ± 445.11 cm/s.

## 4. Discussion

To the best of our knowledge, the present study is an innovative investigation into the long-term effects of SAVR on arterial stiffness, wave reflections, and central hemodynamics using a range of measurements, employing a robust methodological approach. Additionally, it is the first study to examine the effects of SAVR on arterial properties depending on the type of valve used. Furthermore, we explored the potential influence of arterial stiffness on the QOL following SAVR, an area that has been minimally studied in this patient population. The results of our study show that arterial stiffness increases shortly after SAVR and continues to increase significantly over time, regardless of systemic blood pressure or perceived quality of life. In patients with SV prosthesis, the cfPWV may increase more compared to the MV or the BV groups.

### 4.1. Exploring the Pathophysiological Interplay in the SAVR Patient Cohort

#### 4.1.1. Post-Operative Period

During the post-operative period, we observed a significant increase in cfPWV between 4 and 9 days following surgery. This increase in arterial stiffness can be attributed to various aortic surgical procedures, including aortic cannulation, cooling, clamping, incision, and suturing [17,32,38]. The nature of these procedures can compromise the integrity of the aortic wall and lead to temporary changes in its structure [36,37,38]. In addition, the phenomenon of aortic root “stunning” can contribute to localized edema and damage to the vasa vasorum, resulting in the fragmentation of elastic and collagen fibers [39]. These factors collectively contribute to the observed escalation of arterial stiffness. However, it is important to consider that the arterial system may respond to a restoration of AV function with a temporary increase in stiffness to adapt to the altered flow dynamics [32,36].

#### 4.1.2. Arterial Stiffness in the Long Term

In the measurements conducted at one year, we observed that increased aortic stiffness was sustained, as shown by the cfPWV and baPWV. This prolonged increase in aortic stiffness can be attributed to several factors. It is possible that after SAVR, the arterial system eventually adapts to the new hemodynamic conditions by continually intensifying the transmitted load it receives [32]. Aside from the response of the aortic wall to this pressure and volume loading with increased stiffness, other factors such as a further recruitment of collagen fibers may play a role in the long term [40,41].

Several studies have attempted to investigate the vascular hemodynamic changes that occur after aortic valve repair. Most studies present acute results of increased arterial stiffness and suggest that this situation may correlate with possible prognostic implications [14,38,42]. Research by Al-Musa et al. underscores that aortic stiffness remains elevated for six months post-SAVR, indicating that the reason lies in the effects of the surgical procedure on the aortic wall. Manipulation of the aorta can be the cause of increased stiffness that persists beyond the immediate post-operative period and is explained as follows: The SAVR procedure, which involves an aortotomy and disrupts the integrity of the aortic wall, results in damage to the vasa vasorum. This trauma, compounded by the removal of periaortic fat containing the vasa vasorum, acutely exacerbates aortic distensibility, contributing to a persistent increase in arterial stiffness [37]. Similarly, findings from another study reveal a significant rise in aortic stiffness following SAVR, as assessed using tonometry and phase-contrast MRI. Importantly, this increase in arterial stiffness is observed independently of changes in blood pressure, suggesting a direct effect of the surgical procedure on the biomechanical properties of the aorta [40].

In addition, the surgical procedure is accused of triggering an acute surge in flow transmission and increased wall stress in the proximal aorta, leading to increased arterial stiffness. This situation, initiated in the early post-operative period, persists in the long term [36]. A cohort of patients underwent TAVR with long-term results and found that the increase in arterial stiffness was persistent in the long term; it was interpreted that this may be due to an increase in blood pressure caused by the removal of the valve obstruction [32].

In our cohort, a significant increase in arterial stiffness was observed despite similar blood pressure at baseline and at 1 year, suggesting that more mechanisms are involved in the increase in arterial stiffness over the change in blood pressure. With this concept in mind, we conducted further analyses to investigate the potential prognostic significance of variations in arterial stiffness. We found a negative correlation between the change in cfPWV at one-year follow up and both baseline cfPWV and the change in peak aortic valve velocity determined by echocardiography. Baseline cfPWV was found to be a primary predictor of ΔcfPWV, indicating that a lower baseline value is associated with a greater increase in cfPWV over time. This observation suggests a plausible explanation: a higher degree of left ventricular outflow obstruction, leading to a greater reduction in stroke volume, results in significantly improved transmission to the arterial system and, thus, a correspondingly stiffer response to the aorta [32].

However, further research is needed to elucidate the precise relationship between baseline arterial stiffness, changes in arterial properties, and long-term clinical outcomes in patients undergoing SAVR. It is difficult to explain changes in cfPWV by vascular wall remodeling or hemodynamic changes at the LV and ventriculo–aortic transition levels [43]. To clarify the precise interplay of structural remodeling of the vascular wall or changes in hemodynamics and in cfPWV, more investigation is required.

### 4.2. Prosthesis Valve Type: Does It Impact Arterial Stiffness?

To our knowledge, this study is the first to investigate the possible effect of valve type on arterial stiffness after SAVR. In patients who received an SV prosthesis, a doubling in cfPWV values was observed in comparison with those who received MV or BV. This finding implies a possible relationship between the type of valve used and the increase in arterial stiffness.

A reasonable explanation for this finding is that conventional valves include a sewing ring that occupies part of the outer valve surface. In contrast, the design of SVs does not require stitching or a sewing ring, a fact that makes them almost “stentless”. As a result, SVs have a larger effective orifice area compared to conventional valves [44]. Additionally, SVs require a shorter duration of cardiopulmonary bypass pump or aortic cross clamp, leading to an improved hemodynamic response [45]. As a consequence, SVs allow for greater flow transmission, meaning that blood passing through the valve exerts higher pressure on the arterial walls. This heightened pressure and increased flow could potentially contribute to the observed higher arterial stiffness in patients who received this type of valve [46]. However, it is important to acknowledge that further research with larger populations is necessary to establish a definitive causal relationship between valve type and the observed increase in arterial stiffness. Other factors, including patient characteristics and underlying cardiovascular conditions, may also influence the development of arterial stiffness in patients who undergo SV implantation.

### 4.3. Wave Reflections

In response to peripheral hemodynamic changes after SAVR, an initial decrease in Alx@75 is expected, reflecting peripheral vasodilation. On the other hand, the increase in SEVR indicates an improvement in the blood supply to myocardial tissue following AV repair. At one year, the return of Alx@75 to baseline values can be interpreted as the prevailing equilibrium in peripheral vascular resistance within the altered hemodynamic state. On the other hand, the continuous improvement in SEVR suggests that the initial improvement in myocardial tissue perfusion is sustained even after one year [32].

### 4.4. QOL

To take a comprehensive approach, we assessed the QOL status of our study population and conducted sub-analyses to investigate whether the choice of valve type had any impact on our findings or correlated with variations in arterial stiffness. Interestingly, we observed that there were no significant correlations between the three valve types and arterial stiffness indices (cfPWV, baPWV) in terms of PCS or MCS. However, the increase in these two main domains of QOL suggest that SAVR has a positive impact on patients physical functioning, mental well-being, and overall life satisfaction. To date, there has been limited research on the impact of arterial stiffness on the QOL after SAVR. There is one study suggesting that impaired values of aortic stiffness before the procedure could have a negative effect on the QOL in various domains in the post-operative period [27]. This contradictory result to our study findings indicates that the QOL may be influenced by factors other than just aortic stiffness. Further research is needed to understand the underlying mechanisms and the possible association of the QOL with arterial stiffness after SAVR.

## 5. Clinical Implications

This study improves the decision-making process by providing valuable insight into the accurate assessment of hemodynamic stress in the LV. In addition, it identifies patients with poor prognosis who may benefit from interventions to manage arterial stiffness, particularly in the context of high blood pressure.

### 5.1. Capturing the True Hemodynamic Load of the LV

Accurate assessments of LV hemodynamic load in patients with AVS are challenging because the multidimensional nature of the disease may present obstacles to determining vascular and valvular dysfunction. Recent studies have recognized that the use of arterial biomarkers can provide valuable insight into the comprehensive assessment of the true severity of AVS. For instance, valvulo–arterial impedance (Zva), which captures both valvular and vascular components, is one of these biomarkers, and its prognosis has been associated with poor outcomes in AVS. However, Zva alone cannot distinguish between valvular and arterial contributions in patients with paradoxical, severe, low-flow or low-gradient AVS, although it detects cases in which the gradient and blood pressure falsely appear normal [47,48]. This evaluation gap could be closed by directly measuring arterial stiffness. In fact, increased aortic stiffness serves as an indicator of patients at risk of developing paradoxical low-flow and low-gradient subtypes as AVS progresses, independent of echocardiographic severity indices [38]. By incorporating these arterial biomarkers and indices, clinicians can gain a better understanding of the true hemodynamic load in AVS and make more informed decisions in patient management [36].

### 5.2. Prognostic Role

We observed a progressive increase in arterial stiffness post-surgery, persisting at the one-year mark. Interestingly, this increase in arterial stiffness in our sample did not significantly correlate with a decline in the QOL, as the QOL notably improved. It is worth noting that contrasting findings in various studies involving AVS populations and arterial stiffness measurements suggest a potential impact of arterial stiffness on mortality, morbidity, or QOL in those cases. For instance, a recent prospective study of TAVI patients found that invasively estimated PWV was the dominant predictor of 1-year mortality [42]. In other studies, increased baseline cfPWV was a predictor of cardiovascular events and worse prognosis. Another study of asymptomatic patients with moderate AVS showed a strong association between increased baseline cfPWV values, cardiovascular events, and worse prognosis. Furthermore, cfPWV measurements have been identified as independent predictors of NYHA class, cognitive function, and the QOL both before and after surgical intervention [27,36,49,50]. These findings underscore the potential of arterial biomarkers in predicting various clinical endpoints beyond mortality in patients with AVS.

## 6. Limitations

Our study methodology corresponds to studies with the most appropriate design. However, there are some limitations worth considering. Firstly, it is important to note that measurements of arterial stiffness and wave reflections in severe AVS have not yet undergone full validation, despite their utilization in previous studies. Although our findings align with similar studies and indicate that arterial stiffness values are not influenced by AVS obstruction, further validation and standardization of these measurements are warranted. Another limitation is that pharmacological treatment was left to the discretion of the surgeons. Therefore, it may be that the antihypertensive medications changed periprocedurally and during follow-up, influencing our results. Nevertheless, these modifications occurred in a small proportion of our study population. Furthermore, 19 of 60 patients in our study were diagnosed with CAD and had CABG. Although no correlation was found between CAD of CABG and cfPWV fluctuation after controlling for all time points, we cannot be sure that there is no mechanism linking these two parameters in patients undergoing SAVR. Further research with a larger sample is needed to investigate this possible relationship. Finally, as a secondary objective, we explored the correlation between prosthesis valve type and arterial stiffness (cfPWV). However, several limitations were addressed in this attempt. Firstly, the small size of each subgroup did not allow us conduct further analyses (e.g., propensity matching). Secondly, there were differences in the baseline measurements of cfPWV and clinical characteristics among the valve-type groups (e.g., age and baseline SBP), which may have introduced extra confusing effects on changes in cfPWV.

Despite these limitations, our study contributes to the growing body of evidence on arterial stiffness in severe AVS and provides valuable insights into the relationship between arterial stiffness and AVS obstruction. Further research is needed to address these limitations and enhance our understanding of this complex relationship.

## 7. Conclusions

In conclusion, our study offers valuable insights into the influence of SAVR on arterial properties. We observed a significant increase in arterial stiffness one-year post-SAVR. Notably, this rise in arterial stiffness did not show a correlation with a decline in measures of quality of life (QOL). Furthermore, we observed significant increases in arterial stiffness among patients who underwent SAVR, with SV showing a greater effect compared to mechanical and biological valves. Although our research offers a preliminary understanding of the connection between arterial stiffness and prosthesis valve type, more research is necessary to make firm conclusions in this field.

## Figures and Tables

**Table 1:** Baseline characteristics.

	Total Population (N = 60)
Age (years)	70.25 ± 8.76
Sex (M/F)	39 (65)/21 (35)
Height (cm)/Weight (cm)	165/82
BMI (kg/m^2^)	29.13 ± 4.83
BSA (m^2^)	1.929 ± 0.31
eGFR (mL/min/1.73 m^2^)	71.65 ± 21.78
Waist (cm)/Hip (cm)	106.38 ± 12.22/106.58 ± 11.12
Logistic Euroscore II (%)	2.46 ± 2.28
Cardiovascular risk factors	
Hypertension, n (%)	44 (73)
Dyslipidemia, n (%)	36 (60)
Diabetes, n (%)	19 (32)
CAD diagnosis, n (%)	19 (32)
Current smokers, n (%)	21 (30)
Atrial Fibrillation, n (%)	9 (15)
Pre-existing conduction abnormality, n (%)	7 (12)
COPD, n (%)	8 (13)
PCI, n (%)	2 (3)
CKD stages I–III, n (%)	3 (5)
PAD, n (%)	1 (2)
Over three comorbidities	37 (62)
NYHA II-III-IV, n (%)	40 (66)-18 (30)-2 (4)
Procedural Characteristics	
Mechanical valve, n (%)	26 (43)
Biological valve, n (%)	21 (35)
Perceval valve, n (%)	13 (22)
Cabg, n (%)	19 (32)
Number of vessels, 1/2/3, n (%)	13 (22)/5 (8)/1 (2)
Valve size (mm) mechanical, n (%)	18:1 20:1 21:7 22:1 23:12 25:4
Valve size (mm) bio prosthetic, n (%)	19:1 21:9 23:9 25:2
Valve size Perceval, n (%)	Small: 2 Medium: 2 Large: 3 X-Large: 6
Aortic cross clamping time (min)	73.70 ± 27.25
CBP time (min)	93.10 ± 28.50
Pharmacological treatment (N = 60)	
ACE inhibitors/ARBs/Aldosterol Antagonist, n (%)	40 (58)
CCBs, n (%)	15 (22)
BBs, n (%)	49 (71)
Statins, n (%)	40 (58)
Ezetimibe, n (%)	3 (4)
Loop diuretics, n (%)	34 (49)
Thiazide diuretics, n (%)	6 (9)
Potassium-sparing diuretics, n (%)	4 (6)
Antiplatelets, n (%)	29 (42)
Acenocoumarol, n (%)	29 (42)
OAC, n (%)	8 (12)
Duration of hospitalisation (days, min–max)	6 ± 1 (4–9)

Categorical variables are presented as absolute and relative frequencies and continuous variables as mean value ± SD. Non-distributed variables, median value (interquartile range 25–75th percentile). ACE, angiotensin-converting enzyme; ARBs, angiotensin-Il type-1 receptor blockers; BBs, beta-blockers; BMI, body mass index; BSA, body surface area; CAD, coronary artery disease; CCB, calcium channel blockers; CABG, coronary artery bypass graft; CBP, cardiopulmonary bypass; CKD, chronic kidney disease; COPD, chronic obstructive pulmonary disease; NOAC, new oral anticoagulants; NYHA, New York Heart Association; PAD, peripheral artery disease; PCI percutaneous coronary intervention.

**Table 2:** The study population’s changes in hemodynamic indices, vascular biomarkers, and echocardiographic indices during the study (N = 60).

	Pre-SAVR	Post-SAVR	1-Year Post-SAVR	*p* Value *^2^
Aortic and peripheral hemodynamic indices				
Aortic systolic pressure (mm Hg)	136 ± 24	118 ± 17 †	138 ± 19 ‡	<0.001
Aortic diastolic pressure (mm Hg)	79 ± 12	73 ± 13 †	79 ± 11 ‡	0.001
Peripheral systolic pressure (mm Hg)	146 ± 26	139 ± 19 †	150 ± 20 ‡	0.003
Peripheral diastolic pressure (mm Hg)	77 ± 13	73 ± 12 †	79 ± 11 ‡	0.004
Heart rate (bpm)	66 ± 10	82 ± 14 †	68 ± 11 ‡	0.000
Vascular biomarkers				
AIx@75 (%)	31.16 ± 10.22	22.73 ± 12.71 †	30.98 ± 9.47 ‡	<0.001
cfPWV (m/s)	7.67 ± 1.70	8.27 ± 1.92 †	9.29 ± 2.59 †‡	<0.001
SEVR (%)	136.16 ± 30.42	149.25 ± 32.74 †	147.50 ± 30.48 †	0.013
ESP	120 ± 20.9	105.10 ± 15.8 †	120.15 ± 16.19 ‡	<0.001
EjD	325.75 ± 48.89	252.58 ± 49.75 †	280.78 ± 108.29 †‡	<0.001
baPWV (cm/s) *^1^ n = 55	1633.36 ± 429	2014.20 ± 606 †	1867.72 ± 408 †	<0.001
Echocardiographic indices				
AV-Vmax (m/s)	4.26 ± 0.63	2.49 ± 0.485 †	2.34 ± 0.585 †‡	<0.001
AVA (cm^2^)	0.74 ± 0.23	1.67 ± 0.39 †	1.61 ± 0.40 †	<0.001
Indexed AVA (cm^2^/m^2^)	0.39 ± 0.11	0.87 ± 0.21 †	0.83 ± 0.19 †	<0.001
Mean Pressure Gradient (mmHg)	44.20 ± 12.1	13.32 ± 5.38 †	11.63 ± 7.09 †‡	<0.001
Left ventricular ejection fraction (%)	55 ± 9	55.91 ± 8.20	57.16 ± 6.53 †‡	0.027
AVA VTI (cm)	99. 80 ± 17	41.55 ± 10.30 †	46.45 ± 15.27 †‡	<0.001
SV (ml/m^2^)	70.20 ± 22	69.12 ± 20.80	74.13 ± 20.75	0.318
SVi (ml/m^2^)	36.12 ± 11.82	35.48 ± 10.37	37.13 ± 11.58	0.500
PASP (mmHg)	35.48 ± 6.74	32.92 ± 4.04 †	33.92 ± 4.89	0.005
Flow Rate	218.77 ± 72.35	267.87 ± 82.11 †	247.92 ± 54.90 †‡	<0.001

Continuous variables are presented as mean value ± standard deviation; AIx@75: augmentation index adjusted for heart rate at 75 beats per minute; AVA: aortic valve area; SEVR: subendocardial viability ratio; ESP: end systolic pressure; EjD: ejection duration; PWV: pulse wave velocity; *p* values were obtained by analysis of variance. † Statistically significant change compared to pre-SAVR. ‡ Statistically significant change compared to post-SAVR. *^1^ Because only patients that had ABI values ≥ 0.9 in both legs at all time points were included in the analysis. *^2^ *p* value: ANOVA or Friedman test.

**Table 3:** Changes in arterial stiffness indices in correlation with the valve type from pre-SAVR to 1-year post-SAVR (Δ).

Type of Valve	Pre-SAVR	Post-SAVR	1-Year Post-SAVR		*p* Value *^1^
cfPWV (m/s) ^N = 60^				ΔcfPWV ^N = 60^	
Mechanical valve (n = 26)	6.63 ± 1.25	7.32 ± 0.97 †	7.82 ± 1.38 †‡	+1.19 ± 1.72	0.006
Biological valve (n = 21)	8.11 ± 1.24	8.60 ± 2.18	9.85 ± 2.16 †‡	+1.73 ± 1.68	<0.001
Sutureless valve (n = 13)	9.05 ± 1.91	9.63 ± 2.01	11.31 ± 3.41 †	+2.25 ± 3.70	0.19
baPWV (cm/s) ^n = 55^				ΔbaPWV ^n = 55^	
Mechanical valve (n = 26)	1405.08 ± 404	1780.85 ± 502	1634.46 ± 278	+208.16 ± 284.30	
Biological valve (n = 21)	1833.52 ± 385	2014.29 ± 589	2114.76 ± 517.37	+207.88 ± 404.85	
Sutureless valve (n = 13)	1680.08 ± 375	2415 ± 606	1999.46 ± 306	+319.38 ± 445.11	

Continuous variables are presented as mean value ± standard deviation. *^1^ *p* value, ANOVA or Friedman test. † Statistically significant change compared to pre-SAVR. †‡ Statistically significant change compared to post-SAVR.

**Table 4:** Baseline and periprocedural characteristics of SAVR patients (N = 60) according to prosthesis valve type and correlations of each valve type with ΔcfPWV.

Type of Valve *^1^	MV (n = 26)	*p* Value *^3^	BV (n = 21)	*p* Value *^3^	SV (n = 13)	*p* Value *^3^
Baseline characteristics n (%)						
Age	63.23 ± 6.52	0.77 ^r = −0.058^	74.67 ± 6.35	0.52 ^r = 0.147^	77.15 ± 5.36	0.87 ^r = −0.047^
Gender (M/F)	17/9	0.60	12/9	0.57	10/3	0.07
BMI (kg/m^2^) *^2^	30.44 ± 5.52	0.59 ^r = 0.108^	28.46 ± 4.51	0.14 ^r = −0.331^	27.61 ± 3.22	0.58 ^r = −0.166^
Euroscore II (%)	1.49 ± 1.08	0.52 ^r = −0.131^	2.78 ± 2.02	0.85 ^r = 0.044^	3.88 ± 3.47	0.004 ^r = 0.736^
Pre EF (%) *^2^	57.30 ± 7.90	0.007 ^r = −0.517^	55.23 ± 9.14	0.052 ^r = 0.429^	50.38 ± 10.50	0.13 ^r = −0.439^
Pre SBP (mmHg) *^2^	138.88 ± 22.70	0.39 ^r = 0.175^	154.19 ± 29.53	0.26 ^r = 0.255^	147.85 ± 24.53	0.42 ^r = −0.244^
>3 comorbidities	17 (65)	0.45	11 (52)	0.058	9 (69)	0.03
Periprocedural characteristics n (%)						
CABG *^2^	9 (35)	0.78	6 (29)	0.75	4 (31)	0.49
CBP time (min) *^2^	105.81 ± 24.31	0.53 ^r = −0.128^	91.76 ± 27.18	0.24 ^r = −0.264^	69.85 ± 24.36	0.33 ^r = 0.291^
ACC time (min) *^2^	82.73 ± 24.69	0.80 ^r = −0.052^	75.95 ± 25.58	0.19 ^r = −0.297^	52 ± 24.38	0.32 ^r = 0.295^

Continuous variables are presented as mean value ± standard deviation. *^1^ Mechanical valve (MV), Biological valve (BV), sutureless valve (Perceval, SV). *^2^ Body mass index (BMI), ejection fraction (EF), systolic blood pressure (SBP), cardiopulmonary artery bypass graft (CABG), cardiopulmonary bypass pump (CBP), aortic cross clump (ACC). *^3^ *p* values of bivariate or independent sample analysis of each valve group with change from pre-SAVR to 1-year post-SAVR (∆) carotid–femoral pulse wave velocity (ΔcfPWV).

## Data Availability

Data are unavailable due to privacy or ethical restrictions.

## Supplementary Materials

The following supporting information can be downloaded at: https://www.mdpi.com/article/10.3390/jpm14050509/s1, Supplementary Materials S1: Exclusion criteria of the study; Supplementary Materials S2: Assessment of quality of life; Supplementary Materials S3: Periprocedural antihypertensive treatment (N = 60); Supplementary Materials S4: Measurement of peripheral blood pressures, central blood pressures and wave reflection indices; Supplementary Materials S5: Periprocedural complications based the Major Adverse Cardiovascular Events; Supplementary Materials S6: Multivariable regression analysis of the change from pre-SAVR to 1-year post-SAVR carotid-femoral pulse wave velocity (ΔcfPWV) to patient and procedural characteristics; Supplementary Materials S7: Results: Effects of SAVR on peripheral pressures, central hemodynamics and wave reflections; Supplementary Materials S8: Change of peripheral and aortic blood pressures post-SAVR; Supplementary Material S9: Scores for Perceived QOL domains and differences in correlation with change from pre-SAVR to 1-year post-SAVR carotid-femoral pulse wave velocity (ΔcfPWV); Supplementary Materials S10: Changes from pre-SAVR to 1-year post-SAVR (Δ) arterial stiffness indices in correlation with the valve type (Mechanical valve-MV, Biological valve-BV, Sutureless valve-SV). References [51,52,53] are cited in the supplementary materials.
