# Supplementary material for: The Effect of Surgical Aortic Valve Replacement on Arterial Stiffness: Does the Valve Type Matter?
Source: J Pers Med. 2024 May 11;14(5):509. doi: 10.3390/jpm14050509 (PMC11122145; doi:10.3390/jpm14050509)
Supplement: Supplementary file 1 [file jpm-14-00509-s001.zip › jpm-2985822-supplementary.pdf]

Supplementary Materials

Supplementary Materials 1.

Exclusion criteria of the study:

Patients with known history of a bicuspid aortic valve, endocarditis, active neoplastic disease and antineoplastic treatment within the last 6 months, severe left ventricular dysfunction with left ventricular ejection fraction (LVEF) <20%, mixed aortic valve disease (aortic stenosis and aortic regurgitation > Grade II), chronic kidney disease stage IV/V or dialysis patients, pregnancy, peripheral vascular disease, severe dementia were excluded.

Supplementary Materials 2.

Assessment of quality of life:

QOL was assessed using an established instrument in the AVR population (SF 36)[1]. We used the RAND 36-Item Short Form Health Survey (Version 1.0)[2]. It uses a metric rating system from 0 to 100 to measure patient-reported outcomes on aspects of physical and mental health (the highest rate indicates the best possible health). The questions are divided into eight health domains: physical functioning (PF), role functional/physical (RP), bodily pain (BP), general health (GH), energy/fatigue (Vitality-VT), social functioning (SF), Role Emotional-well being (RE), and mental health (MH). RP, BP, GH and VT consist of the summary of physical components (PCS) and VT, SF, RE and MH consist of the summary of mental components (MCS). Responses were scored as described by the RAND 36-Item Health Survey (Version 1.0).

Supplementary Materials 3.

Table S1. Periprocedural antihypertensive treatment (N=60).

Antihypertensive medication	Pre SAVR (n, %)	Post SAVR (n, %)
Diuretics	34 (49)	42 (61)
Calcium Channel Blockers	16 (23)	15 (22)
Angiotensin Converting Enzyme Inhibitors or Angiotensin II Receptor Blockers	35 (51)	40 (58)
B-blockers	37 (54)	49 (71)

Supplementary Materials 4.

Measurement of peripheral blood pressures, central blood pressures and wave reflection indices:

At each session an applanation tonometer was placed over the right radial and the aortic pulse waveform, which was derived from the radial waveform using a transfer function, was recorded. In the row data, the best 3 measurements with a satisfactory operator index above 80% were entered. Recordings with bigeminy, trigeminy, rapid atrial fibrillation, or isolated premature ventricular or atrial beats and the following compensatory beats were excluded from the analysis.

Central pressures and waveforms were calculated based on the values of the brachial peripheral pressures as recorded. Augmentation pressure (AP=systolic pressure minus pressure at the inflection point of the aortic waveform), is the additional pressure in the reflected wave returning to the heart that increases the SBP and hence LV workload. After the AP calculation, the augmentation index (AIx) was calculated and presented as a percentage. A correction was made for the corrected heart rate index of 75 bpm (AIx@75) because Alx is significantly affected by heart rate. Finally, the subendocardial viability ratio index (SEVR) was calculated based on systolic and diastolic pressure and their time integrals[21] and is expressed as the ratio of aortic diastolic area to aortic systolic area under the curve of the central aortic pulse wave.

Supplementary Materials 5.

Table S2. Periprocedural complications based the Major Adverse Cardiovascular Events[3] (MACE, N=60).

MACE (in-hospital and 30 days)	n, (%)
Atrial fibrillation	11 (19)
Acute kidney injury*	10 (17)
Implantation of permanent pacemaker	4 (7)
Re-Intubation	1 (2)
Transfusions*	12 (37)

Categorical variables are presented as absolute and relative frequencies. *Acute Kidney Injury-AKI, defined according KDIGO criteria as SerCr≥0.3mg/dl in the first 6-12h, no patient underwent renal dialysis, * Transfusions: red blood transfusions due to anaemia without an evident bleeding

Supplementary Materials 6.

Table S3. Multivariable regression analysis of the change from pre-SAVR to 1-year post-SAVR carotid-femoral pulse wave velocity (ΔcfPWV) to patient and procedural characteristics.

	Model 1			Model 2		
	Beta	t	*P*	*Beta*	t	*P*
Change of echocardiographic peak aortic valve velocity from baseline to 1-year post-SAVR (Vmax, m/s)	-0.0362	-3.013	0.004	-0.369	-2.966	0.005
Baseline cfPWV (m/s)	-0.518	-3.563	0.001	-0.517	-3.488	0.001
Age (years)	0.68	0.387	0.70	0.400	0.400	0.69
Sex (female/male)	-0.112	-0.927	0.35	-0.129	-1.043	0.30
Bioprosthetic Valve	0.236	1.405	0.16	0.233	1.359	0.18
Sutureless Valve	0.370	2.019	0.049	0.201	1.422	0.16
Peripheral SBP at 1 year in Model 1 (mmHg)	0.244	1.983	0.053			
Central SBP at 1 year in Model 2 (mmHg)				0.174	1.395	0.16

Adjusted R^2 of model 1= 0.226   Adjusted R^2 of model 2= 0.197

cfPWV; Carotid-femoral pulse wave velocity; SBP, systolic blood pressure

*Mechanical valve excluded

Supplementary Materials 7.

Results: Effects of SAVR on peripheral pressures, central hemodynamics and wave reflections.

Peripheral systolic BP (SBP) and diastolic BP (DBP) had differences over the analysis period (p= 0.003 and p=0.004 respectively, Figure S2). More specifically, after the procedure SBP showed a statistically significant difference compared to baseline (146 ± 26 mmHg vs 139 ± 19 mmHg, p=0.042 and 77 ± 13 mmHg vs 73 ± 12 mmHg, p= 0.048 for SBP and DBP, respectively). At 1-year, peripheral pressures were found unchanged compared to baseline (146 ± 26 mmHg vs 150 ± 20 mmHg, p=.25 and 77 ± 13 mmHg vs 79 ± 11 mmHg, p=.25) and increased compared to measurements at discharge (139 ± 19 mmHg vs 150 ± 20 mmHg, p=.001 and 73 ± 12 vs 79 ± 11, p=0.001 for SBP and DBP respectively).

As for central pressures, during follow-up statistically significant fluctuations were observed in both aortic SBP (p<.001) and aortic DBP (p=.001). Immediately after SAVR aortic SBP was found decreased compared to baseline values (136 ± 24 mmHg vs 118 ± 17 mmHg, p=.010) and similarly, aortic DBP (79 ± 12 mmHg vs 73 ± 13 mmHg, p=.014). At 1 year follow-up, compared to baseline measurements, central pressures were found unchanged (136 ± 24 mmHg vs 138 ± 19 mmHg, p=.627 for aoSBP and 79 ± 12 mmHg vs 79 ± 11 mmHg, p=.710 for aoDBP) but similarly to peripheral pressures, there was a statistical significance comparing the measurements between the acute phase and 1-year measurements (118 ± 17 mmHg vs 138 ± 19 mmHg, p<.001 and 73 ± 13 mmHg vs 79 ± 11 mmHg, p=.002 for aoSBP and aoDBP respectively).

Supplementary Materials 8.

Figure S1. Change of peripheral and aortic blood pressures post-SAVR.

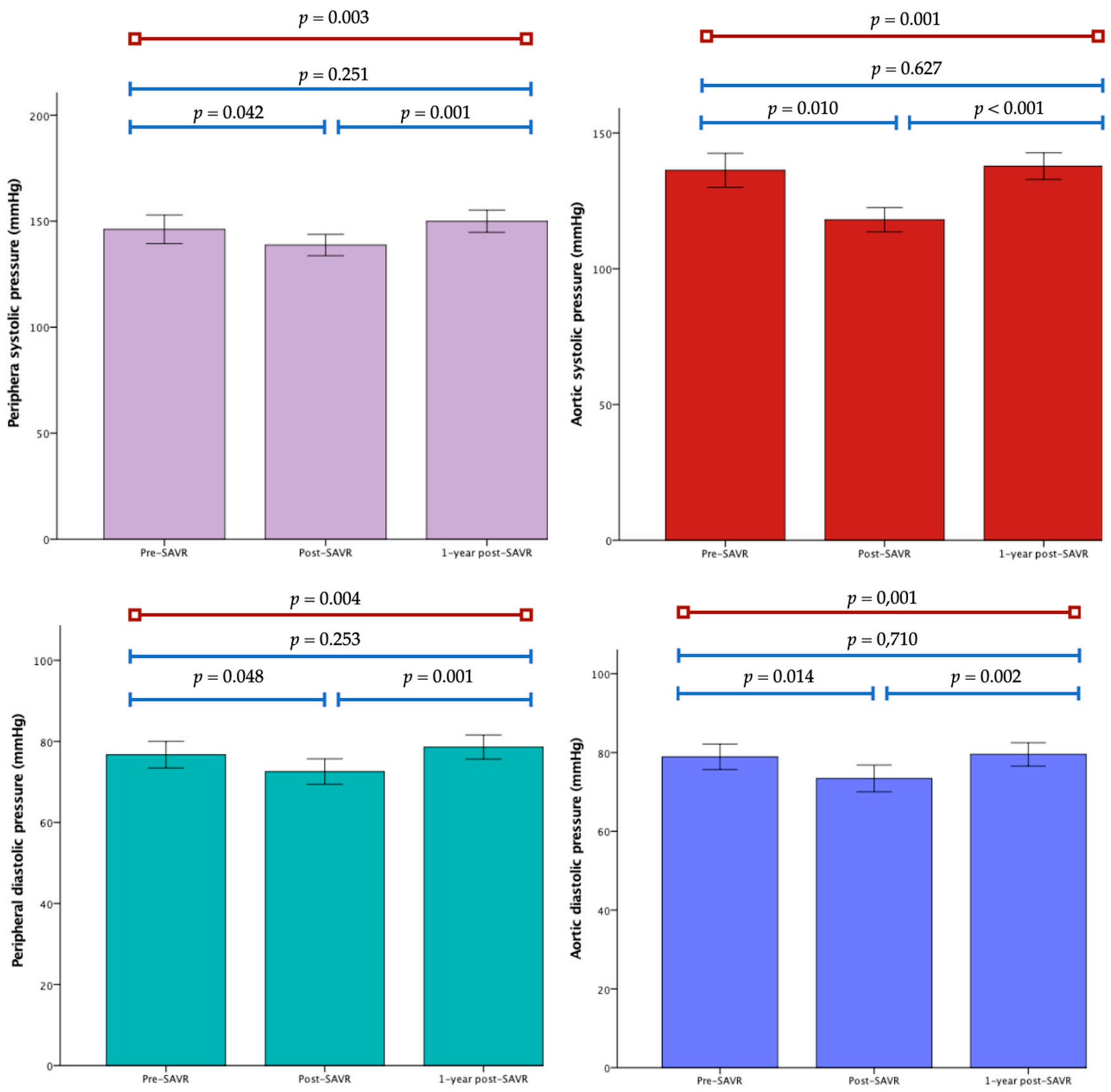

P values were obtained by analysis of variance and paired-samples analysis. SBP, systolic blood pressure; DBP, diastolic blood pressure.

Supplementary Material 9.

Table S4. Scores for Perceived QOL domains and differences in correlation with change from pre-SAVR to 1-year post-SAVR carotid-femoral pulse wave velocity (ΔcfPWV) at 1-year-post SAVR (N=60, SF-36).

QOL	Items	Pre-SAVR	1-year-post SAVR	P-value*2	Mean difference	ΔcfPW Correlation*3	Significance of correlation*3
SF-36*1 (%)							
**Physical functioning**	10	50 (36-64)	80 (75-85)	<.001	+25 (15-45)	0.117	0.37
**Role functional / physical**	4	50 (40-50)	82 (75-85)	<.001	+35 (25-50)	0.046	0.72
**Role emotional**	3	50 (35-50)	90 (85-100)	<.001	+50 (50-60)	0.039	0.76
**Energy/fatigue**	4	55 (46-55)	75 (65-80)	<.001	+20 (10-35)	-0.086	0.51
**Emotional well-being**	5	68 (55-84)	77 (72-88)	.001	+8 (-4.7-25)	0.038	0.77
**Social functioning**	2	55 (50-55)	75 (60-80)	<.001	+20 (20-25)	0.124	0.34
**Pain**	2	66 (38.5-68)	80 (70-90)	<.001	+24 (4.12-50)	0.112	0.39
**General health**	5	65 (45-65)	80 (75-90)	<.001	+25 (15-40)	0.248	0.056
**Health change**	1	65 (51-65)	90 (75-100)	<.001	+30 (10-38)	-0.027	0.83
**PCS*1**		56 (40-62)	80 ± 9.6	<.001	ΔPCS +24 (17-44)	0.158	0.22
**MCS*1**		55 (50-61)	80±1.0	<.001	ΔMCS +25 ± 12	0.003	0.98

Continuous variables are presented as mean value ± Standard Deviation; Non distributed variables, Median value (Interquartile range 25th-75th percentile).
*1 Short Form 36 questionnaire (SF 36), Physical component summary (PCS), Mental component summary (MCS).
*2 P-value: Paired-samples analysis between mean value differences of preoperative to 1-year post-SAVR PCS, MCS.
*3 P-value: Bivariate analysis analysis between change from pre-SAVR to 1-year post-SAVR carotid-femoral pulse wave velocity (ΔcfPWV) and mean scores and differences of SF-36.

Supplementary Materials 10.

Figure S2. Changes from pre-SAVR to 1-year post-SAVR (Δ) arterial stiffness indices in correlation with the valve type (Mechanical valve-MV, Biological valve-BV, Sutureless valve-SV)

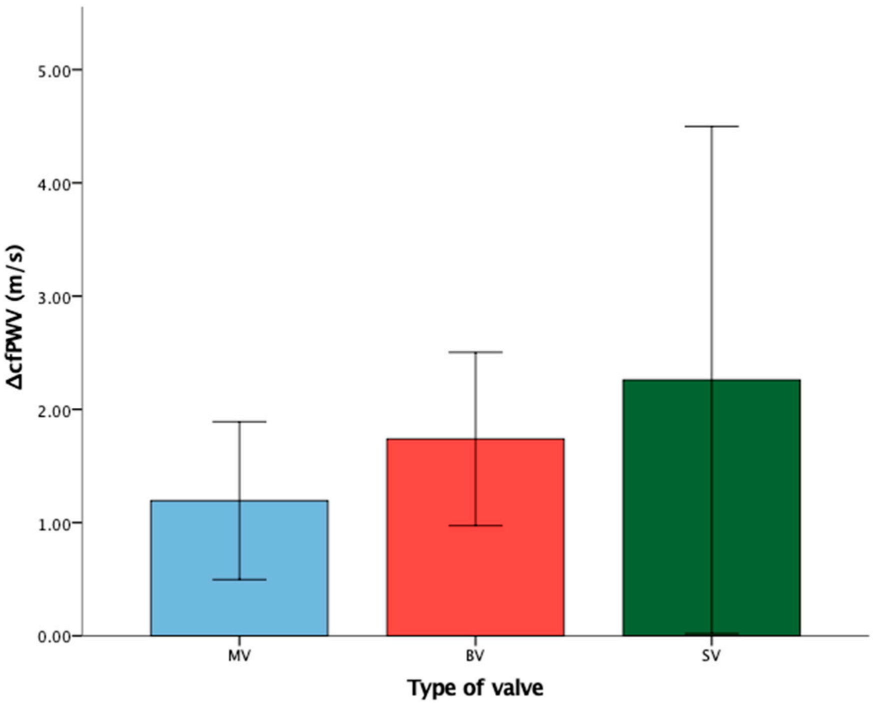

References

(1) Rex, C. E.; Heiberg, J.; Klaaborg, K. E.; Hjortdal, V. E. Health-related quality-of-life after transapical transcatheter aortic valve implantation. *Scand Cardiovasc J* 2016, *50* (5-6), 377-382. DOI: 10.1080/14017431.2016.1235725.
(2) Saimanen, I.; Kuosmanen, V.; Rahkola, D.; Selander, T.; Kärkkäinen, J.; Harju, J.; Aspinen, S.; Eskelinen, M. RAND-36-Item Health Survey: A Comprehensive Test for Long-term Outcome and Health Status Following Surgery. *Anticancer Res* 2019, *39* (6), 2927-2933. DOI: 10.21873/anticanres.13422.
(3) Banovic, M.; Putnik, S.; Penicka, M.; Doros, G.; Deja, M. A.; Kockova, R.; Kotrc, M.; Glaveckaite, S.; Gasparovic, H.; Pavlovic, N.; et al. Aortic Valve Replacement Versus Conservative Treatment in Asymptomatic Severe Aortic Stenosis: The AVATAR Trial. *Circulation* 2022, *145* (9), 648-658. DOI: 10.1161/CIRCULATIONAHA.121.057639.
